# Individual- and community-level neighbor relationships and physical activity among older Japanese adults living in a metropolitan area: a cross-sectional multilevel analysis

**DOI:** 10.1186/s12966-018-0679-z

**Published:** 2018-05-25

**Authors:** Satoshi Seino, Akihiko Kitamura, Mariko Nishi, Yui Tomine, Izumi Tanaka, Yu Taniguchi, Yuri Yokoyama, Hidenori Amano, Miki Narita, Tomoko Ikeuchi, Yoshinori Fujiwara, Shoji Shinkai

**Affiliations:** Research Team for Social Participation and Community Health, Tokyo Metropolitan Institute of Gerontology, 35-2 Sakae, Itabashi, Tokyo, 173-0015 Japan

**Keywords:** Physical activity, Sitting time, Sedentary behavior, Social relationships, Social capital, Older adults, Multilevel analysis

## Abstract

**Background:**

Informal neighbor relationships (NRs) are considered a structural aspect of social relationships. Although NRs might affect physical activity (PA), no previous study has simultaneously examined compositional and contextual associations of NRs with PA. In this study, we examined whether individual- and community-level NRs were independently associated with PA.

**Methods:**

We analyzed cross-sectional data from 8592 (4340 men and 4252 women) non-disabled residents aged 65–84 years from all 18 districts of Ota City, Tokyo. PA was assessed by using the International Physical Activity Questionnaire-Short Form. In addition, we calculated moderate-to-vigorous PA (MVPA), its components (vigorous PA [VPA], moderate PA [MPA], and walking time [WT]), and sitting time (ST). Individual-level NRs were categorized as “visiting each other,” “standing and chatting,” “exchange of greetings,” or “none.” Community-level NRs were defined as the proportions of residents with active NRs (i.e., those in the categories visiting each other and standing and chatting) in the 18 districts. Using multilevel regression analyses, we examined independent associations of individual- and community-level NRs with PA variables and adjusted for important confounders.

**Results:**

Individual-level NRs were consistently positively associated with MVPA and its components (VPA, MPA [in men], and WT) in both sexes, and the dose–response relationships were significant (all *P* < 0.041 for trend). In men, community-level NRs (by 1% estimation) were positively associated with individual MVPA (2.1 metabolic equivalent-hours/week, 95% confidence interval: 0.7–3.4), VPA (8.6 min/week, 2.7–14.4), and WT (11.6 min/week, 2.2–20.9), regardless of the degree of individual-level NRs. Significant cross-level interactions of NRs with MVPA and VPA were observed among men, and the dose–response relationships were significant (both *P* < 0.037 for trend). Neither individual- nor community-level NRs were associated with ST in either sex.

**Conclusions:**

Men and women with inaccessible neighbors engaged in less MVPA, while men living in communities with active NRs engaged in more MVPA, regardless of individual-level NRs. NRs at the individual and community level might help prevent physical inactivity among men.

## Background

Physical inactivity is an important contributor to non-communicable diseases and—after high blood pressure, tobacco use, and high blood glucose—is the fourth most important risk factor for mortality [1, 2]. Among older adults, physical activity (PA) includes recreational or leisure-time activity, transportation, occupation, household chores, play, games, sports, and planned exercise in the context of daily, family, and community activities [1]. Regular PA helps maintain and improve physical and cognitive function among older adults [3–5]. Despite growing evidence of the health benefits of PA and the presence of established recommendations for healthy aging [1, 6], physical inactivity remains a major concern for older and younger adults [7]. Thus, attention has increasingly focused on public health efforts designed to increase PA. In Japan, a new initiative, Healthy Japan 21 (second term)—a 10-year national health promotion campaign to extend healthy life expectancy and reduce health disparities by establishing targets in 53 specific areas [8]—was launched in 2013 by the Ministry of Health, Labor and Welfare. Increasing PA is one of its explicit goals.

Recent public health studies have examined the social connection offered by relationships, as social relationships can affect health behaviors such as PA [9] and outcomes such as mortality [10–12] and are likely to promote healthy communities. Social relationships are broadly defined as the degree to which individuals are interconnected and embedded in communities [13] and have both structural and functional aspects. The structural aspects include social integration, which refers to an individual’s overall level of involvement with informal social relationships (e.g., having a spouse), formal social relationships (e.g., those with volunteer organizations), and the social network, which refers to the web of social relationships surrounding an individual, particularly those structural features such as the type and strength of each social relationship [11]. The functional aspects include social support, which refers to the support exchanged through social relationships [11]. Because these aspects of social relationships can be analyzed at the individual and group level, studies should consider them simultaneously when examining their effects on health behaviors.

A previous systematic review [9] found that people with greater social support for PA (especially when it originated from family members) were more likely to engage in leisure-time PA and highlighted the importance of friends as sources of support for leisure-time PA in older adults. Moreover, a previous study of Japanese older adults reported that active relationships with neighbors might ameliorate infrequent eating habits caused by limited access to food [14]. These findings suggest that informal neighbor relationships (NRs) could affect individual health behaviors through pathways such as social support. However, to our knowledge, no previous study has simultaneously investigated the individual-level (compositional) and community-level (contextual) effects of NRs on PA.

Using data from older adults living in a metropolitan area of Japan, we examined whether individual- and community-level NRs were positively associated with PA. Specifically, we examined if each individual- and community-level NR was independently associated with PA and if observed associations differed by sex.

## Methods

### Study population

We used baseline data from a community-wide intervention study on preventing and reducing frailty in Ota City, Tokyo, Japan (Ota Genki Senior Project), which was launched in 2016 [15]. The full details of the participant selection process were previously published [15]. Briefly, 15,500 residents aged 65–84 years—approximately 10% of the elderly population of Ota City—were selected by using stratified and random sampling strategies in all 18 districts. All participants were physically and cognitively independent, which was defined as absence of long-term care insurance certification.

**Fig. 1:**
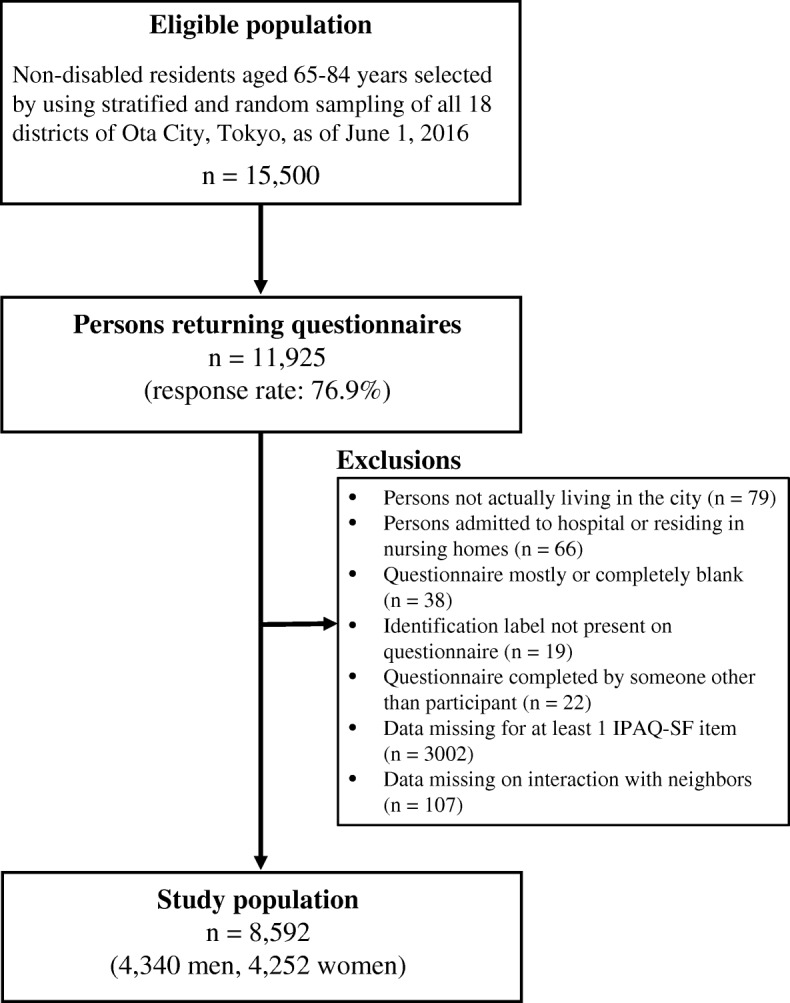
Flow diagram of study participants. IPAQ-SF = International Physical Activity Questionnaire-Short Form

Of the 15,500 self-administered questionnaires distributed in July 2016, 11,925 were returned (response rate, 76.9%) [15]. After excluding 79 questionnaires from respondents who did not actually live in Ota City, 38 questionnaires that were mostly or completely blank, 19 questionnaires with missing identification labels, 22 questionnaires that were completed by someone other than the participant, 66 questionnaires from hospital inpatients and nursing home residents, and 3109 questionnaires with missing information on PA and/or NRs, a total of 8592 questionnaires (4340 men and 4252 women) were ultimately included in our analysis (Fig. 1). The mean district response rate was 77.1% (range, 71.8–80.8%), and the number of respondents per district was 255 to 2135 (mean, 477).

### Measurements

#### PA

PA was evaluated with the Japanese version of the International Physical Activity Questionnaire-Short Form (IPAQ-SF), the external reliability and validity of which have been reported [16, 17]. The IPAQ-SF includes separate items on time spent on vigorous PA (VPA), moderate PA (MPA), walking time (WT), and sitting time (ST) during a typical week. Using these values, we defined total moderate-to-vigorous PA (MVPA) as 7 days × (8.0 metabolic equivalents [METs] × VPA hours/day + 4.0 METs × MPA hours/day + 3.3 METs × walking hours/day) [18]. ST indicates usual weekday ST, apart from sleeping [19].

#### Individual- and community-level NRs

NR was measured by using the following item: “What kind of relations do you have with people in your neighborhood?” [14] The possible answers were “visiting each other,” “standing and chatting,” “exchange of greetings,” or “none.” We used these categories to categorize individual-level NRs. Community-level NRs were created in accordance with the district units by aggregating individual responses in each district. The proportion of people with an active relationship with neighbors (i.e., the categories visiting each other and standing and chatting) in each district was defined as community-level NRs.

#### Covariates

The covariates included age, living situation (living with others or alone), duration of residence in the neighborhood (1–29, 30–59, or ≥ 60 years), educational attainment (junior high school, high school, or junior college/vocational college/college/graduate school graduate), equivalent income (< 2.0, 2.0–3.99, ≥4.0 million yen, or unknown), alcohol drinking and tobacco smoking statuses (current, never, or former), body mass index (< 18.5, 18.5–24.9, or ≥ 25 kg/m^2^), number of chronic diseases (0, 1, or ≥ 2), mobility limitation (presence or absence), self-rated health (excellent to good or fair to poor), depressive mood (presence or absence), employment (yes or no), and social activity (presence or absence). To assess the practical living standard of household members, equivalent income was calculated by dividing household income by the square root of the number of household members [20]. Body mass index was defined as self-rated body weight (kg) divided by self-rated height squared (m^2^). Number of chronic diseases was defined as the sum of the presence of hypertension, hyperlipidemia, cardiovascular disease, cerebrovascular disease, and diabetes mellitus. Mobility limitation was defined as self-reported difficulty in walking one-quarter of a mile (0.4 km) or climbing 10 steps without resting [21]. Depressive mood was evaluated with the five-item short form of the Geriatric Depression Scale (range, 0–5) [22], and the presence of depression was defined as a score of 2 or greater. Participation in any of the following activities more than once a month was defined as presence of social activity: volunteering, civic action, nonprofit organizations, sports groups, hobby and learning groups, senior citizen clubs, neighborhood associations, or others, but excluding employment. If participants did not respond to the covariates, corresponding observations were assigned to the “missing” categories.

### Statistical analyses

All data were analyzed in relation to sex by using Stata 14.2 (StataCorp, TX, USA). The chi-square and unpaired *t* tests were used to compare individual- and community-level NRs between sexes. To examine whether PA variables were systematically associated with NRs, we used a trend test that extended the Wilcoxon rank-sum test (the “nptrend” command in Stata). The chi-square test was used for other characteristic variables. Because our data had a multilevel structure comprising individuals (at level 1) nested within 18 districts (at level 2), we performed multilevel regression analyses with fixed slopes and random intercept models adjusted for all covariates and calculated partial regression coefficients and 95% confidence intervals (CIs) to identify both the individual-level (compositional) and community-level (contextual) effects of NRs on PA variables. MVPA and its components (VPA, MPA, and WT) and ST were defined as the dependent variables. Individual- and community-level NRs, and their cross-level interaction terms and covariates, were defined as fixed factors. The district was defined as a random factor. A linear trend test was used to assess the dose–response relationship between NR level and PA variables. To avoid issues related to multicollinearity, community-level NRs were centered on their grand mean. An *α* of 0.05 was considered to indicate statistical significance.

## Results

Among 4340 men, 14.9% described their NR status as visiting each other, 30.3% as standing and chatting, 44.3% as exchange of greetings, and 10.5% as none. Among the 4252 women, the responses were 29.8%, 44.6%, 22.1%, and 3.6%, respectively. Overall, women were significantly more likely than men to have active NRs at the individual level (*P* < 0.001). The mean proportions and standard deviations of people with active NRs (i.e., the categories visiting each other and standing and chatting) in the 18 districts were also significantly higher for women (74.8 ± 2.7%; range, 67.6–81.5%) than for men (45.6 ± 3.0%; range, 37.7–56.4%) (*P* < 0.001).

Tables 1 and 2 show the PA variables and other participant characteristics, in relation to NR category, for men (Table 1) and women (Table 2). NRs were positively associated with MVPA, VPA, MPA, and WT and inversely associated with ST in men and women (all *P* < 0.027 for trend). As compared with men who selected the category visiting each other, men with no NRs were younger, more likely to be living alone, and had lived in the neighborhood for less time. They were also more likely to be underweight or obese, had lower equivalent incomes, were less likely to drink alcohol, were less likely to be employed, had less social activity, and had more chronic diseases, more mobility limitations, and more depressive moods and poorer self-rated health (Table 1). Women with no NRs were more likely to be living alone, had lived in the neighborhood for less time, had a lower educational level and equivalent incomes and less chronic disease, were less likely to be employed, and had less social activity, more mobility limitations and depressive moods, and poorer self-rated health.

**Table 1:** PA variables and other characteristics of male participants, by category of individual-level NRs

Variables	Category of NRs								*P-value*
	Visiting each other		Standing and chatting		Exchange of greetings		None		
	(*n* = 645, 14.9%)		(*n* = 1317, 30.3%)		(*n* = 1923, 44.3%)		(*n* = 455, 10.5%)		
PA, median (interquartile range)									
MVPA, MET-hours/week	31.8	(13.2–71.1)	27.8	(9.9–59.6)	23.1	(8.3–49.5)	15.4	(2.2–38.6)	< 0.001^b^
VPA, min/week	0	(0–180)	0	(0–120)	0	(0–60)	0	(0–0)	< 0.001^b^
MPA, min/week	0	(0–180)	0	(0–120)	0	(0–75)	0	(0–0)	< 0.001^b^
WT, min/week	300	(150–600)	280	(120–600)	240	(90–480)	180	(0–420)	< 0.001^b^
ST, min/day	270	(180–480)	300	(180–480)	300	(180–480)	360	(180–480)	< 0.001^b^
Age (years), mean (standard deviation)	74.4	(5.3)	74.3	(5.2)	73.9	(5.6)	72.8	(5.5)	< 0.001^b^
Living alone, n (%)	91	(14.1)	173	(13.1)	291	(15.1)	130	(28.6)	< 0.001
Years of residence in the neighborhood, n (%)									< 0.001
1–29	98	(15.2)	191	(14.5)	411	(21.4)	153	(33.6)	
30–59	272	(42.2)	635	(48.2)	876	(45.6)	188	(41.3)	
60-	270	(41.9)	476	(36.1)	621	(32.3)	108	(23.7)	
Education, n (%)									0.132
Junior high school graduation	141	(21.9)	315	(23.9)	385	(20.0)	106	(23.3)	
High school graduation	199	(30.9)	412	(31.3)	615	(32.0)	136	(29.9)	
Junior college/vocational college/college/graduate school graduation	289	(44.8)	549	(41.7)	881	(45.8)	197	(43.3)	
Other/missing	16	(2.5)	41	(3.1)	42	(2.2)	16	(3.5)	
Equivalent income, n (%)									< 0.001
< 2.0 million	221	(34.3)	492	(37.4)	687	(35.7)	164	(36.0)	
2.0–3.99 million	198	(30.7)	454	(34.5)	607	(31.6)	125	(27.5)	
≥ 4.0 million≥4.0 million	152	(23.6)	222	(16.9)	384	(20.0)	84	(18.5)	
Unknown/missing	74	(11.5)	149	(11.3)	245	(12.7)	82	(18.0)	
Alcohol drinking status, n (%)									0.022
Current	476	(73.8)	928	(70.5)	1397	(72.7)	298	(65.5)	
Never or former	165	(25.6)	377	(28.6)	515	(26.8)	150	(33.0)	
Smoking status, n (%)									0.101
Current	102	(15.8)	260	(19.7)	374	(19.5)	107	(23.5)	
Never or former	536	(83.1)	1046	(79.4)	1533	(79.7)	344	(75.6)	
Body mass index, kg/m^2^									0.012
< 18.5	27	(4.2)	56	(4.3)	92	(4.8)	29	(6.4)	
18.5–24.9	474	(73.5)	947	(71.9)	1363	(70.9)	293	(64.4)	
≥ 25	142	(22.0)	310	(23.5)	457	(23.8)	126	(27.7)	
Number of chronic diseases, n (%)^a^									0.027
0	144	(22.3)	244	(18.5)	370	(19.2)	102	(22.4)	
1	202	(31.3)	416	(31.6)	561	(29.2)	126	(27.7)	
2+	248	(38.5)	556	(42.2)	869	(45.2)	185	(40.7)	
Mobility limitation, n (%)	129	(20.0)	301	(22.9)	501	(26.1)	145	(31.9)	< 0.001
Self-rated health, n (%)									< 0.001
Excellent to good	503	(78.0)	1012	(76.8)	1447	(75.3)	304	(66.8)	
Fair to poor	103	(16.0)	214	(16.3)	379	(19.7)	123	(27.0)	
Depressive mood, n (%)	143	(22.2)	396	(30.1)	694	(36.1)	218	(47.9)	< 0.001
Employment, n (%)	309	(47.9)	507	(38.5)	796	(41.4)	156	(34.3)	< 0.001
Social activity, n (%)	345	(53.5)	519	(39.4)	653	(34.0)	95	(20.9)	< 0.001

*PA* physical activity, *NRs* neighbor relationships, *MVPA* moderate-to-vigorous physical activity, *VPA* vigorous physical activity, *MPA* moderate physical activity, *WT* walking time, *ST* sitting time

^a^Sum of the presence of hypertension, hyperlipidemia, cardiovascular disease, cerebrovascular disease, and diabetes mellitus

^b^*P* for trend test

**Table 2:** PA variables and other characteristics of female participants, by category of individual-level NRs

Variables	Category of NRs								*P-value*
	Visiting each other		Standing and chatting		Exchange of greetings		None		
	(*n* = 1268, 29.8%)		(*n* = 1895, 44.6%)		(*n* = 938, 22.1%)		(*n* = 151, 3.6%)		
PA, median (interquartile range)									
MVPA, MET-hours/week	29.7	(13.2–62.7)	26.4	(11.9–57.8)	23.1	(8.3–55.8)	11.6	(0–34.7)	< 0.001^b^
VPA, min/week	0	(0–100)	0	(0–60)	0	(0–20)	0	(0–0)	< 0.001^b^
MPA, min/week	0	(0–120)	0	(0–120)	0	(0–105)	0	(0–0)	< 0.001^b^
WT, min/week	300	(160–630)	350	(150–630)	280	(120–630)	180	(0–420)	< 0.001^b^
ST, min/day	300	(180–480)	300	(180–480)	300	(180–480)	420	(240–600)	0.027^b^
Age (years), mean (standard deviation)	74.0	(5.5)	73.7	(5.4)	73.0	(5.7)	73.9	(6.0)	< 0.001^b^
Living alone, n (%)	339	(26.7)	409	(21.6)	222	(23.7)	60	(39.7)	< 0.001
Years of residence in the neighborhood, n (%)									< 0.001
1–29	169	(13.3)	327	(17.3)	253	(27.0)	54	(35.8)	
30–59	822	(64.8)	1144	(60.4)	497	(53.0)	68	(45.0)	
60-	268	(21.1)	414	(21.9)	182	(19.4)	28	(18.5)	
Education, n (%)									0.008
Junior high school graduation	287	(22.6)	389	(20.5)	216	(23.0)	47	(31.1)	
High school graduation	569	(44.9)	851	(44.9)	386	(41.2)	63	(41.7)	
Junior college/vocational college/college/graduate school graduation	364	(28.7)	607	(32.0)	310	(33.1)	35	(23.2)	
Other/missing	48	(3.8)	48	(2.5)	26	(2.8)	6	(4.0)	
Equivalent income, n (%)									0.012
< 2.0 million	481	(37.9)	652	(34.4)	325	(34.7)	62	(41.1)	
2.0–3.99 million	338	(26.7)	545	(28.8)	241	(25.7)	30	(19.9)	
≥ 4.0 million	206	(16.3)	288	(15.2)	145	(15.5)	17	(11.3)	
Unknown/missing	243	(19.2)	410	(21.6)	227	(24.2)	42	(27.8)	
Alcohol drinking status, n (%)									0.244
Current	538	(42.4)	785	(41.4)	378	(40.3)	48	(31.8)	
Never or former	721	(56.9)	1098	(57.9)	551	(58.7)	101	(66.9)	
Smoking status, n (%)									0.213
Current	64	(5.1)	117	(6.2)	71	(7.6)	6	(4.0)	
Never or former	1191	(93.9)	1757	(92.7)	856	(91.3)	142	(94.0)	
Body mass index, kg/m^2^									0.056
< 18.5	117	(9.2)	236	(12.5)	124	(13.2)	17	(11.3)	
18.5–24.9	893	(70.4)	1310	(69.1)	652	(69.5)	102	(67.6)	
≥ 25	246	(19.4)	339	(17.9)	152	(16.2)	30	(19.9)	
Number of chronic diseases, n (%)^a^									0.038
0	302	(23.8)	493	(26.0)	259	(27.6)	43	(28.5)	
1	393	(31.0)	583	(30.8)	289	(30.8)	41	(27.2)	
2+	457	(36.0)	686	(36.2)	301	(32.1)	48	(31.8)	
Mobility limitation, n (%)	391	(30.8)	552	(29.1)	313	(33.4)	66	(43.7)	< 0.001
Self-rated health, n (%)									< 0.001
Excellent to good	1010	(79.7)	1492	(78.7)	710	(75.7)	95	(62.9)	
Fair to poor	186	(14.7)	316	(16.7)	182	(19.4)	46	(30.5)	
Depressive mood, n (%)	306	(24.1)	630	(33.3)	384	(40.9)	86	(57.0)	< 0.001
Employment, n (%)	339	(26.7)	466	(24.6)	287	(30.6)	34	(22.5)	< 0.001
Social activity, n (%)	667	(52.6)	904	(47.7)	364	(38.8)	26	(17.2)	< 0.001

*PA* physical activity, *NRs* neighbor relationships, *MVPA* moderate-to-vigorous physical activity, *VPA* vigorous physical activity, *MPA* moderate physical activity, *WT* walking time, *ST* sitting time

^a^Sum of the presence of hypertension, hyperlipidemia, cardiovascular disease, cerebrovascular disease, and diabetes mellitus

^b^*P* for trend test

Tables 3 and 4 show associations of individual- and community-level NRs with PA variables, as determined by multilevel regression analyses after adjusting for all covariates, in men (Table 3) and women (Table 4). Among men, individual-level NRs were consistently positively associated with MVPA and its components VPA, MPA, and WT, and the dose–response relationships were significant (all *P* < 0.041 for trend). Community-level NRs (by 1% estimation) were positively associated with male MVPA (2.1 MET-hours/week; 95% CI, 0.7–3.4), VPA (8.6 min/week; 2.7–14.4), and WT (11.6 min/week; 2.2–20.9). Significant cross-level interactions between individual- and community-level NRs were observed for male MVPA, VPA, and WT. The dose–response relationship was significant for MVPA (*P* = 0.025 for trend) and VPA (*P* = 0.037 for trend) but not for WT (*P* = 0.072 for trend).

**Table 3:** Multilevel regression analyses of associations between individual- and community-level NRs and PA variables in men (*n* = 4340)

Variables	MVPA, MET-hours/week			VPA, min/week			MPA, min/week			WT, min/week			ST, min/day		
	B	95% CI	*P*	B	95% CI	*P*	B	95% CI	*P*	B	95% CI	*P*	B	95% CI	*P*
Fixed effects															
Individual-level NRs															
Visiting each other	Ref.			Ref.			Ref.			Ref.			Ref.		
Standing and chatting	−4.4	(−9.6, 0.8)	0.098	−22.4	(−45.2, 0.5)	0.055	− 21.4	(−47.2, 4.4)	0.104	0.6	(−36.0, 37.2)	0.975	19.6	(−3.5, 42.7)	0.097
Exchange of greetings	−6.0	(−11.0, −1.0)	0.018	−20.0	(−41.9, 1.9)	0.074	−25.0	(−49.8, −0.3)	0.047	−30.3	(−65.5, 4.8)	0.091	16.8	(−5.4, 39.1)	0.138
None	−11.8	(−18.7, −4.9)	0.001	−36.9	(−67.1, −6.7)	0.017	−46.7	(−80.8, −12.7)	0.007	−68.0	(−116.3, −19.6)	0.006	15.8	(−15.2, 46.7)	0.318
	Trend		0.001	Trend		0.041	Trend		0.012	Trend		0.001	Trend		0.355
Community-level NRsaCommunity-level NRs^a^															
1% estimation	2.1	(0.7, 3.4)	0.002	8.6	(2.7, 14.4)	0.004	4.2	(−2.4, 10.8)	0.216	11.6	(2.2, 20.9)	0.015	−3.9	(−9.9, 2.0)	0.196
Cross-level interaction term															
Visiting each other × community-level NRs	Ref.			Ref.			Ref.			Ref.			Ref.		
Standing and chatting × community-level NRs	−1.5	(−3.2, 0.1)	0.073	−6.6	(−13.9, 0.6)	0.073	−0.6	(−8.8, 7.6)	0.887	−10.7	(−22.3, 0.9)	0.071	−0.9	(−8.3, 6.5)	0.810
Exchange of greetings × community-level NRs	−1.6	(−3.2, −0.1)	0.046	− 6.3	(−13.2, 0.7)	0.076	−2.0	(−9.8, 5.9)	0.623	−11.7	(− 22.8, −0.6)	0.039	1.4	(−5.7, 8.5)	0.696
None × community-level NRs	−2.5	(−4.6, −0.4)	0.018	−10.7	(−19.9, −1.5)	0.022	−5.5	(−15.8, 4.9)	0.300	−13.4	(−28.1, 1.3)	0.075	2.8	(−6.5, 12.2)	0.550
	Trend		0.025	Trend		0.037	Trend		0.301	Trend		0.074	Trend		0.362
Random effects															
Community-level variance (standard error)	0.00	(0.00)		10.23	(46.35)		0.00	(0.00)		0.00	(0.00)		0.00	(0.00)	
Intraclass correlation coefficient	0.0000			0.0002			0.0000			0.0000			0.0000		

*PA* physical activity, *NRs* neighbor relationships, *MVPA* moderate-to-vigorous physical activity, *METs* metabolic equivalent, *VPA* vigorous physical activity, *MPA* moderate physical activity, *WT* walking time, *ST* sedentary time, *B* partial regression coefficient, *CI* confidence interval

All models were adjusted by age, living situation, years of residence in the neighborhood, education, equivalent income, alcohol drinking status, smoking status, body mass index, number of choronic diseases, mobility limitation, self-rated health, depressive mood, employment status, and social activity

^a^The proportions of people with active interaction (visiting each other and standing and chatting) in districts were calculated for community-level NRs

**Table 4:** Multilevel regression analyses of associations between individual- and community-level NRs and PA variables in women (*n* = 4252)

Variables	MVPA, MET-hours/week			VPA, min/week			MPA, min/week			WT, min/week			ST, min/day		
	B	95% CI	*P*	B	95% CI	*P*	B	95% CI	*P*	B	95% CI	*P*	B	95% CI	*P*
Fixed effects															
Individual-level NRs															
Visiting each other	Ref.			Ref.			Ref.			Ref.			Ref.		
Standing and chatting	−3.1	(−6.8, 0.6)	0.100	−17.3	(−32.6, −1.9)	0.028	−5.8	(−23.8, 12.1)	0.525	−6.3	(−36.9, 24.4)	0.688	13.6	(−3.9, 31.2)	0.127
Exchange of greetings	−5.1	(−9.5, −0.6)	0.026	−17.6	(−36.2, 1.0)	0.064	1.6	(−20.1, 23.4)	0.883	−55.7	(−92.9, −18.6)	0.003	3.5	(−17.8, 24.7)	0.748
None	−18.8	(−27.8, −9.8)	< 0.001	−44.6	(−82.0, −7.1)	0.020	−50.5	(−94.4, −6.7)	0.024	−159.2	(−233.3, −85.1)	< 0.001	39.2	(−4.4, 82.8)	0.078
	Trend		< 0.001	Trend		0.009	Trend		0.320	Trend		< 0.001	Trend		0.252
Community-level NRsaCommunity-level NRs^a^															
1% estimation	0.2	(−0.9, 1.3)	0.767	0.2	(−4.2, 4.6)	0.930	0.7	(−4.5, 5.8)	0.800	0.6	(−8.2, 9.5)	0.539	−2.1	(−7.2, 2.9)	0.403
Cross-level interaction term															
Visiting each other × community-level NRs	Ref.			Ref.			Ref.			Ref.			Ref.		
Standing and chatting × community-level NRs	−0.2	(−1.6, 1.1)	0.769	−2.8	(−8.5, 2.8)	0.321	−1.4	(−8.0, 5.2)	0.678	4.7	(−6.5, 16.0)	0.406	2.3	(−4.1, 8.6)	0.488
Exchange of greetings × community-level NRs	−0.1	(−1.7, 1.4)	0.863	0.0	(−6.5, 6.5)	0.998	0.1	(−7.6, 7.7)	0.982	−2.4	(−15.5, 10.6)	0.713	0.3	(−7.1, 7.8)	0.929
None × community-level NRs	−1.4	(−4.6, 1.9)	0.406	−4.9	(−18.5, 8.7)	0.484	−1.1	(−17.0, 14.8)	0.894	−13.3	(−40.2, 13.6)	0.333	2.6	(−13.2, 18.5)	0.747
	Trend		0.657	Trend		0.734	Trend		0.986	Trend		0.527	Trend		0.823
Random effects															
Community-level variance (standard error)	5.65	(6.44)		0.00	(0.00)		0.00	(0.00)		0.00	(0.00)		0.00	(0.00)	
Intraclass correlation coefficient	0.0022			0.0000			0.0000			0.0000			0.0000		

*PA* physical activity, *NRs* neighbor relationships, *MVPA* moderate-to-vigorous physical activity, *METs* metabolic equivalent, *VPA* vigorous physical activity, *MPA* moderate physical activity, *WT* walking time, *ST* sedentary time, *B* partial regression coefficient, *CI* confidence interval

All models were adjusted by age, living situation, years of residence in the neighborhood, education, equivalent income, alcohol drinking status, smoking status, body mass index, number of choronic diseases, mobility limitation, self-rated health, depressive mood, employment status, and social activity

^a^The proportions of people with active interaction (visiting each other and standing and chatting) in districts were calculated for community-level NRs

Among women, individual-level NRs were positively associated with MVPA and its components VPA and WT, and the dose–response relationships were significant, as was the case for men (all *P* < 0.009 for trends). However, the associations between community-level NRs and PA variables and cross-level interactions were not significant. Neither individual nor community-level NRs were significantly associated with ST in either sex.

The range of interclass correlation coefficients was 0.0000 to 0.0022 for PA variables in both sexes, which indicated that variances in PA variables, as explained by the random effects for districts, were extremely small (0.00–0.22%).

## Discussion

The present sex-stratified multilevel analyses revealed three main findings. First, individual-level NRs were consistently positively associated with MVPA and its components VPA and WT in both sexes, and the dose–response relationships were significant. Second, in men, community-level NRs were positively associated with individual MVPA, VPA, and WT, regardless of the degree of individual-level NRs. Finally, significant cross-level interactions between individual- and community-level NRs were observed in PA variables for men.

An association of regular PA with mortality has been reported [23, 24]. The Japanese PA Reference for Health Promotion 2013 [25] recommends PA of 23 MET-hours/week or more at age 18–64 years and 10 MET-hours/week or more at age 65 years or older. Furthermore, the official Japanese PA guidelines for health promotion (the “ActiveGuide”) [26] suggest an additional 10 min of daily PA to increase healthy life expectancy for all generations. A large prospective cohort study [27] reported that 15 min per day or 90 min per week of moderate-intensity exercise resulted in a 14% lower risk of all-cause mortality and increased life expectancy by 3 years, even among individuals at risk for cardiovascular disease. Our findings showed a difference of approximately 6 MET-hours/week (equivalent to 90 min MPA per week) for men and 13 MET-hours/week (equivalent to 195 min MPA per week) for women between respondents who reported an NR status of none and those who reported exchange of greetings. In addition, male MVPA increased by 2.1 MET-hours/week (equivalent to 31.5 min MPA per week) for each 1% increase in community-level NRs. These findings suggest that, in men, activating NRs at the individual and community level contributes to meeting PA recommendations [25–27].

The mechanism by which individual-level NRs enhance MVPA can be explained by a model proposed by Berkman and Krishna [13]. According to this model, social networks are embedded in larger sociocultural contexts, including culture, socioeconomic factors, and politics. Their model also holds that such upstream forces influence network structure and the characteristics of network ties (i.e., NRs in this study). Next, these networks operate at the behavioral level by following primary pathways (i.e., downstream factors), namely, provision of social support, social influence, social engagement, person-to-person contact, access to resources and material goods, and negative social interactions (including conflict and abuse) [13]. Among these pathways, the provision of social support and social engagement may, as compared with other pathways, have a greater direct contribution to MVPA.

A noteworthy finding of this study was that the contextual effects of NRs on PA variables and cross-level interactions were observed only in men. The presence of significant cross-level interaction indicates that men with inactive NRs living in communities in which NRs were inactive had even lower levels of MVPA than did those living in communities in which NRs were active. As to why this was not observed in women, most older women may have already been embedded in more diverse, and larger, informal networks than were men, as was reported in previous studies [28, 29]. Indeed, while 74.4% of women had active NRs in this study, this was the case for only 45.2% of men. This may explain the sex differences in the contextual effects of NRs.

Our findings might also be attributable to the patriarchal values underlying Japanese society (e.g., Japan ranked 157th among 193 countries in female representation in the national parliament, as of 1 December 2017) [30]. The present study area had 217 neighborhood associations in 18 districts, and 68.5% of older adults were members of the 217 neighborhood associations in 2017 [31]. Characteristically, the leaders and executives of these neighborhood associations were almost always men. The neighborhood associations perform various voluntary regional activities in cooperation with the local administration and promote daily exchange of social support among residents. Interestingly, previous studies reported that Japanese men were more likely than women to benefit from interventions that increase social networks [32] and benefited more than women from bonding social capital (i.e., resources accessed within networks or groups in which the members share similar background characteristics, such as sex) [33]. Because the present community-level unit of analysis was the district, NRs from male leaders or executives in the neighborhood associations (i.e., social support between men) could have easily directly influenced the MVPA of male inhabitants.

Our study has several limitations. First, the self-administered questionnaire used for measurement may be subject to recall bias. Although the IPAQ-SF is recommended and widely used for easy assessment of PA, it tends to overestimate reported PA, as compared with objective devices [17]. Second, selection bias is a concern, as 3002 (approximately 25%) of the respondents were excluded because of missing IPAQ-SF items. Third, our findings were obtained by analyzing data from older adults living in a metropolitan area in Japan. Thus, differences between countries and regions (urban or rural) should be considered when extrapolating these data to other settings. Finally, the direction of causality cannot be inferred in a cross-sectional study. We plan to conduct follow-up surveys, to examine longitudinally the associations observed in this study.

Despite these limitations, this study is to our knowledge the first to report associations of NRs with MVPA and its domains among older adults. Moreover, our evidence is strengthened by the use of a multilevel modeling approach to simultaneously examine the compositional and contextual effects of NRs.

In conclusion, although the compositional effects of NRs on PA were observed in both sexes, contextual effects and their cross-level interactions were observed only in men. An approach promoting NRs at both the individual and community level may therefore be important, particularly for preventing physical inactivity among men.

## Abbreviations

CIs: Confidence intervals
IPAQ-SF: International Physical Activity Questionnaire-Short Form
METs: Metabolic equivalents
MPA: Moderate physical activity
MVPA: Moderate-to-vigorous physical activity
NRs: Neighbor relationships
PA: Physical activity
ST: Sitting time
VPA: Vigorous physical activity
WT: Walking time
